# Viability of Collagen Matrix Grafts Associated with Nanohydroxyapatite and Elastin in Bone Repair in the Experimental Condition of Ovariectomy

**DOI:** 10.3390/ijms242115727

**Published:** 2023-10-29

**Authors:** Renato de Moraes, Ana Maria de Guzzi Plepis, Virgínia da Conceição Amaro Martins, Claudio Fernandes Garcia, Ewerton Alexandre Galdeano, Fernanda Latorre Melgaço Maia, Eduardo Gomes Machado, Marcelo de Azevedo e Souza Munhoz, Daniela Vieira Buchaim, Victor Augusto Ramos Fernandes, Rodrigo Alves Beraldo, Rogerio Leone Buchaim, Marcelo Rodrigues da Cunha

**Affiliations:** 1 Interunit Postgraduate Program in Bioengineering (EESC/FMRP/IQSC), University of São Paulo (USP), São Carlos 13566-590, Brazil; renatoijot@gmail.com (R.d.M.); amplepis@iqsc.usp.br (A.M.d.G.P.); claudiofgarcia@alumni.usp.br (C.F.G.); marcelocunha@g.fmj.br (M.R.d.C.); 2 São Carlos Institute of Chemistry, University of São Paulo, USP, São Carlos 13566-590, Brazil; virginia@iqsc.usp.br; 3 Department of Morphology and Pathology, Medical College of Jundiai, Jundiaí 13202-550, Brazil; eagaldeano@gmail.com (E.A.G.); eduardomachado@g.fmj.br (E.G.M.); marcelomunhoz@g.fmj.br (M.d.A.e.S.M.); victorfernandes@g.fmj.br (V.A.R.F.); rodrigo.beraldo@g.fmj.br (R.A.B.); 4 Department of Implant Dentistry, Faculdade São Leopoldo Mandic, Campinas 13045-755, Brazil; felatorrem@gmail.com; 5 Medical School, University Center of Adamantina (UniFAI), Adamantina 17800-000, Brazil; danibuchaim@alumni.usp.br; 6 Postgraduate Program in Structural and Functional Interactions in Rehabilitation, University of Marilia (UNIMAR), Marilia 17525-902, Brazil; 7 Graduate Program in Anatomy of Domestic and Wild Animals, Faculty of Veterinary Medicine and Animal Science, University of Sao Paulo, Sao Paulo 05508-270, Brazil; 8 Department of Biological Sciences, Bauru School of Dentistry (FOB/USP), University of Sao Paulo, Bauru 17012-901, Brazil

**Keywords:** collagen, elastin, nanocompounds, hydroxyapatite, polymers, bone repair, bone regeneration

## Abstract

Bone lesions have the capacity for regeneration under normal conditions of the bone metabolism process. However, due to the increasing incidence of major traumas and diseases that cause bone-mineral deficiency, such as osteoporosis, scaffolds are needed that can assist in the bone regeneration process. Currently, natural polymeric scaffolds and bioactive nanoparticles stand out. Therefore, the objective of the study was to evaluate the osteoregenerative potential in tibiae of healthy and ovariectomized rats using mineralized collagen and nanohydroxyapatite (nHA) scaffolds associated with elastin. The in-vivo experimental study was performed with 60 20-week-old Wistar rats, distributed into non-ovariectomized (NO) and ovariectomized (O) groups, as follows: Controls (G1-NO-C and G4-O-C); Collagen with nHA scaffold (G2-NO-MSH and G5-O-MSH); and Collagen with nHA and elastin scaffold (G3-NO-MSHC and G6-O-MSHC). The animals were euthanized 6 weeks after surgery and the samples were analyzed by macroscopy, radiology, and histomorphometry. ANOVA and Tukey tests were performed with a 95% CI and a significance index of *p* < 0.05. In the histological analyses, it was possible to observe new bone formed with an organized and compact morphology that was rich in osteocytes and with maturity characteristics. This is compatible with osteoconductivity in both matrices (MSH and MSHC) in rats with normal conditions of bone metabolism and with gonadal deficiency. Furthermore, they demonstrated superior osteogenic potential when compared to control groups. There was no significant difference in the rate of new bone formation between the scaffolds. Ovariectomy did not exacerbate the immune response but negatively influenced the bone-defect repair process.

## 1. Introduction

Severe bone injuries can cause disability, compromise quality of life and can hardly be repaired naturally by the body [1], requiring scaffolds that simulate the bioactivity of the extracellular matrix (ECM) and offer temporary support for bone regeneration [2,3]. Although the autologous graft presents unparalleled advantages for regenerative therapies, tissue engineering has improved polymeric scaffolds to overcome weaknesses, such as the risk of premature resorption, osteonecrosis and immunological rejection, and thus, it can offer new therapeutic perspectives, avoiding additional surgical procedures and donor-site morbidity [4,5,6]. Biopolymers have advantageous characteristics for clinical applicability due to their availability, mechanical resistance, bioactivity, biocompatibility and degradability, which can be controlled in acidic environments [7,8]. However, the combination of two or more components may be necessary to achieve the properties necessary to mimic the ECM and stimulate phenotypic expression [9].

Scaffolds for the recovery of bone defects can be made up of metal alloys, [10] bioceramics, synthetic components of polyesters and polyurethanes [7], or natural components such as polysaccharides and proteins [11]. There is a growing process of investigation into proteins naturally present in bones, such as collagen and elastin [12,13,14,15,16]. Collagen is secreted by fibroblasts, being abundant for type I, while types III and V are present in smaller quantities, and together, they make up heterotypic fibrils in the interstitial portion of the ECM. The fibrils are organized in the form of a scaffold within an amorphous gel composed of fibronectin and elastin, which contribute to the nanostructured and three-dimensional organization [16].

Bioabsorbable collagen scaffolds can aid in biochemical signaling and wound healing [16] due to the hemostatic effects present even when implanted under unfavorable clinical conditions [17,18]. Collagen is the most abundant protein in the human body and makes up 80% of all protein content in bones. It is structured by polypeptide chains containing amino acids that interact through covalent bonds that intertwine with the chains [19]. In the initial phase of self-assembly, hydroxylations of proline and lysine residues and glycated bonds to hydroxyproline residues occur [20]. Subsequently, post-translational modifications begin through the transient integration of the molecular protector and the domains of the procollagen chain through disulfide bonds, giving the triple-helical molecular form [21]. 

Maturation is characterized by the removal of non-collagenous propeptides by procollagen amino and carboxy proteases, originating an alpha chain measuring 1.25 nm wide and 300 nm long with a mass of 285 kDa [22]. Five of these parallel chains form a microfibril 1 cm long and 1 mm in diameter with 67 nm bands, where the deposition of hydroxyapatite, the main inorganic constituent of the osteoid matrix secreted by osteoblasts, subsequently occurs [23]. Despite significant advances in collagen synthesis and purification processes, there is still no scaffold that fully reproduces the properties of the native protein; there are some minimal differences remaining in its structure [24] and it is susceptible to hydrolytic and enzymatic degradation if it comes into contact with body fluid [25]. The proteins also have low osteoinductivity [26], which favors premature resorption and the loss of mechanical stability [27], and for these reasons, other components are often added to increase useful life and resistance and obtain greater osteogenic potential [25,26,27].

An alternative for osteoconduction is the addition of hydroxyapatite, a bioactive ceramic based on calcium phosphate [28] with a structure similar to biological hydroxyapatite, and it represents 65% of the inorganic composition of bone tissue [29]. This composite has been used for decades, and its osteogenic capacity and biocompatibility are well-established in the literature [28,29,30]. In contrast to collagen, hydroxyapatite has high crystallinity and low solubility, factors that influence the rapid adhesion and early fixation of the implant to the recipient bed, making the interposition of connective tissue and, consequently, the reabsorption of the implant difficult. Due to this characteristic, it is also not recommended to use this ceramic in isolation [31]; it is more effective when combined with natural or synthetic polymers [32], or even when it is customized into nanoparticles for applications in multicomponent scaffolds [33]. This allows it to obtain better mechanical properties and is more favorable for cell recognition, osteoconduction and reducing the risk of infections [34].

Nanohydroxyapatite (nHA) is considered a reinforcement technology for scaffolds capable of improving resistance and promoting greater biological activity at the bone–implant interface [33]. The possibility of providing nanoroughness to the surface of the scaffolds represents a differential for osteointegration, hydrophilic and piezoelectric potential [29,30,31,32,33,34,35]. Currently, some collagen and hydroxyapatite biomaterials are regulated and demonstrate results close to those of autologous grafts [27,28,29,30,31,32,33,34,35,36]. However, larger bone defects or tissues with metabolic and mineral deficiency require scaffolds with greater biomimetic capacity and functionality. In this context, mineralized collagen scaffolds with nHA have demonstrated controlled biodegradation and positive effects for accelerating osteogenesis [37,38] when applied to healthy organisms or under pathological conditions.

One of the unfavorable conditions for bone remodeling is osteoporosis—a metabolic, systemic and progressive disease mainly associated with aging and estrogen deficiency after menopause [39,40]. Osteoporosis is characterized by the inhibition of the activity of osteoblasts and growth factors, such as: bone morphogenetic protein (BMP), platelet-derived growth factor (PDGF), vascular endothelial growth factor (VEGF), insulin-like growth factors 1 (IGF-1) and fibroblast growth factor 2 (FGF-2) [35]. The low activity of these factors predisposes individuals to a reduction in collagen synthesis and apatite deposition, with osteoclast activity prevailing. The reduction in mineral density causes bone fragility and increases the risk of fractures, morbidity and mortality, representing a problem for global public health given the 9 million fractures treated annually [41].

Conversely, few studies have been carried out on the applicability of scaffolds with sufficient bioactivity and conductivity to overcome the deleterious effects of the disease [42,43]. To improve the performance of scaffolds, it is necessary to enhance angiogenesis and cell signaling pathways by adding osteoprogenitor cells, drugs, growth factors [36] or proteins that are naturally present in bone tissue [27,28,29,30,31,32,33,34,35,36,37,38,39,40,41,42,43,44] to the scaffold. Among proteins of natural origin, elastin also stands out, as it is a constituent of the ECM that presents biological activity and is non-toxic and stable in the long term [45].

Elastin synthesis occurs from tropoelastin, a soluble precursor synthesized by fibroblasts and, through its hydrophobic domains, is associated by covalent bonds with various components of the ECM, such as proteoglycans, fibulins and latent binding protein TGF-β-4, and this process is called coacervation [15,16,17,18,19,20,21,22,23,24,25,26,27,28,29,30,31,32,33,34,35,36,37,38,39,40,41,42,43,44,45,46]. Then, the tropoelastin and lysine precursors undergo oxidative deamination by enzymes from the lysyl oxidases family, resulting in the formation of lysine, and finally, the cross-linking phase begins in which reactions occur between the remaining lysine domains with lysine, or there is aldol condensation between two lysin residues [47], forming an elastin core covered by microfibrils composed of glycoproteins, with type I and II fibrils being predominant [48].

Furthermore, elastin has fibronectin domains and arginine, glycine and aspartic acid (RGB) amino acid sequences, which stimulate differentiation, cell adhesion and angiogenesis [45] in addition to modulating coagulation [49]. Despite the risk of incomplete decellularization in the process of manufacturing elastin polymers, this composite receives attention due to the wide range of biochemical advantages, variety of obtaining and processing methods, and the possibility of composing natural polymeric matrices [50,51]. The synthesis of natural polymers may be economically unfeasible and therefore extraction from mammals is normally chosen [52]. Collagen can be obtained from bovine or porcine tendons, bones and pericardia, with porcine collagen xenografts presenting better prospects due to biocompatibility and similarities with human collagen [53]. Elastin is present in most connective tissues, mainly in the walls of blood vessels, ligaments, lungs, skin, heart valves and cartilage [54], and like collagen, elastin can be purified through alkaline hydrolysis and freeze-drying [55]. As for nHa, it is possible to produce it synthetically from calcium phosphates [56].

This evidence supports the manufacture of natural and mineralized polymeric scaffolds for applications in critical bone defects and under different clinical conditions. Therefore, this present study aimed to determine the osteoconductive potential of polymeric scaffolds made from collagen derived from porcine serosa, mineralized with nanohydroxyapatite and supplemented with elastin from bovine auricular cartilage. The scaffolds were grafted into rats with a healthy skeletal system and into rats with bone-mineral deficiency caused by ovariectomy.

## 2. Results and Discussion

### 2.1. Scaffolds

**Figure 1:**
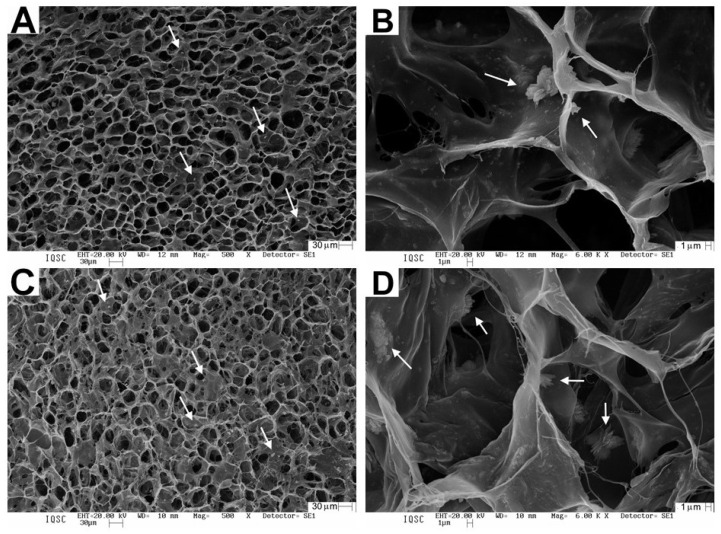
Photomicrographs of the matrices: (**A**) MSH 500×; (**B**) MSH 6000×; (**C**) MSHC 500×; (**D**) MSHC 6000×. Arrows indicate nHA clusters.

The photomicrographs show matrices with a porous surface (Figure 1A,C), and at higher magnifications, the presence of nHA clusters is observed (Figure 1B,C).

Table 1 (below) shows the pore sizes of the scaffolds.

**Figure 2:**
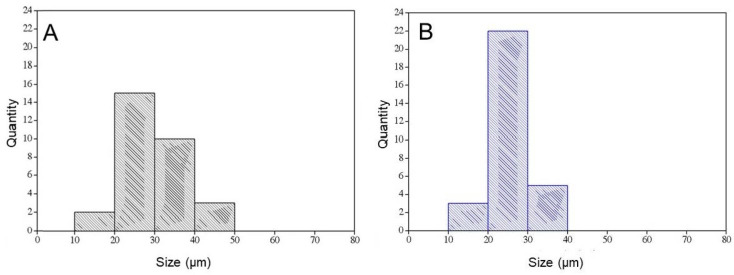
Histograms of the pore sizes of the matrices: (**A**) MSH; (**B**) MSHC.

Figure 2 shows the histograms of the pore sizes of the matrices: (A) MSH; (B) MSHC.

**Figure 3:**
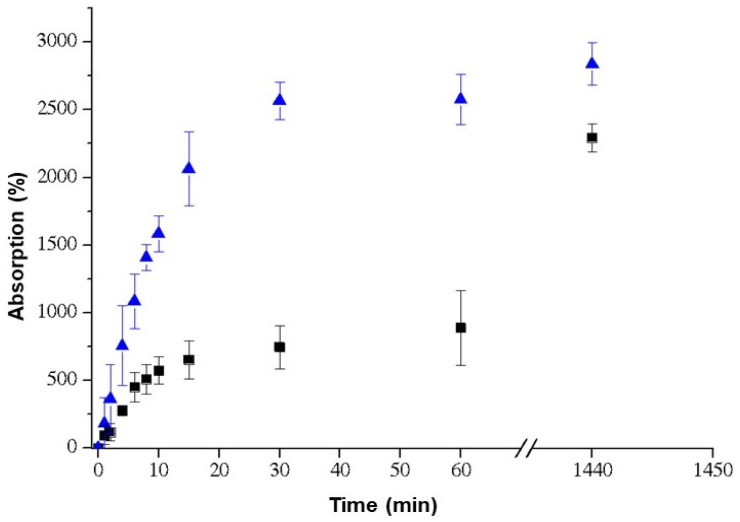
PBS absorption (%): (◼) MSH; (▲) MSHC.

Cell growth involves several factors, including pore size. The pore size required for neovascularization to occur is 5 µm, and the penetration of fibrous tissue requires pores between 10 and 75 µm [57]. The denaturation temperature of the MSH scaffold was 44.0 °C and the presence of nHA and auricular cartilage did not modify this value. The transition observed in DSC occurs due to heating and is generated by the transition of the collagen triple helix to a structure in which the cross-links of the tertiary structure are broken. Thus, there is a transition from a highly organized structure to a disorganized one, which is gelatin [58,59]. Figure 3 and Table 2 shows the uptake kinetics of phosphate-buffered saline into the matrices. It is observed that the MSH matrix presents the smallest and slowest absorption, obtaining 2290% in 24 h, while the matrices containing cartilage (MSHC) showed greater swelling, with absorption being 2837%. Although auricular cartilage has a hydrophobic chemical structure due to the presence of elastin [60], this hydrophobic effect was not observed in relation to the MSH matrix.

### 2.2. In-Vivo Macroscopic and Radiological Analysis

This present study evaluated the osteoconductive potential of natural polymeric scaffolds made of collagen from the serosa of the porcine intestine mineralized with nanohydroxyapatite and supplemented or not with elastin derived from bovine auricular cartilage. They were implanted in bone defects in the tibia of rats under normal conditions of osteometabolism and in rats with bone-mineral deficiency caused by bilateral ovariectomy. For this purpose, we hope that the results presented can offer new perspectives for the development of biomaterials, with an osteogenic potential capable of overcoming the clinical limitations caused by diseases such as osteoporosis.

The clinical applicability of natural collagen and elastin polymers is still restricted due to a limited understanding of the mechanisms of interaction with cells and other components of the ECM [61,62]. Natural polymers derived from animal sources require additional care in the procurement and processing processes, due to the risk of disease transmission and unwanted immunological response. In the case of bovine collagen, thrombin is one of the main components that can cause inflammatory processes [63]. Despite the limitations in the use of xenografts [64], studies demonstrate that natural polymers have greater potential to stimulate cellular differentiation when they are implanted in bones of endochondral origin [65,66].

**Figure 4:**
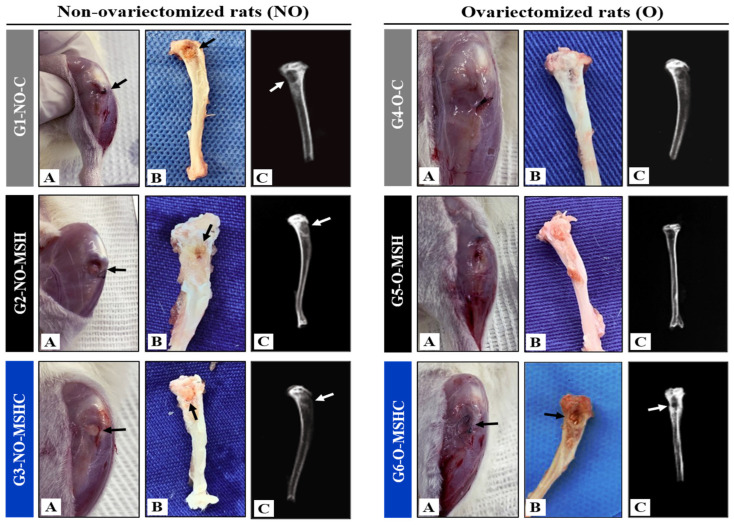
Groups of non-ovariectomized rats on the left and groups of ovariectomized rats on the right. (**A**) In-vivo macroscopic images; (**B**) Macroscopic images of the dissected tibiae; (**C**) Radiological images of the tibias. Arrows indicate the area of the bone defect.

The macroscopic analysis of this study revealed the expected healing of six weeks after surgery to create the experimental defect. In none of the study groups were signs of inflammatory processes observed, given the clinical conditions of the subcutaneous soft tissues with perfusion and absence of adhesions, as shown in images A and B in Figure 4.

In the case of the tibia, a bone of endochondral origin, the bone repair process begins with an important local sterile inflammatory reaction, observable by the presence of a hematoma on the edges of the defect and in the bone marrow cavity. In this hematoma, the formation of fibrous and cartilaginous granulation tissue occurs, which is then mineralized due to the intense activity of osteoblasts [67]. Under normal conditions of osteometabolism, this natural inflammatory process cannot exceed two weeks [68].

Contributing to the literature, no persistent edemas were identified in the areas of the bone defect. Both in the healthy groups and in the groups with ovariectomy, the areas grafted with porcine serosal collagen membranes mineralized with nHA (G2-NO-MSH and G5-O-MSH) and with the membranes supplemented with cartilage-derived elastin bovine ear tissue (G3-NO-MSHC and G6-O-MSHC) showed well-defined edges and a discreet presence of blood fluids around the biomaterials. This clinical finding is a positive indicator regarding the stimulation of angiogenesis in the bone defect. The inability to provide blood supply represents one of the main factors causing osteonecrosis, and consequently, scaffold failure [69].

No other signs suggestive of infectious complications were observed. Gonadal hormone deficiency also did not intensify the local inflammatory response in the diseased control group and in the grafted groups (G4-O-C, G5-O-MSH and G6-O-MSHC). The clinical results observed in the experimental groups were compatible with those identified in the control groups (G1-NO-C and G4-O-C), indicating that the scaffolds did not exacerbate the inflammatory response. These findings confirm the affinity of collagen with the ECM in in-vivo therapies [17]. It is worth mentioning that the experimental protocol adopted in this study applied the principles of the ARRIVE guidelines of the National Center for the Replacement, Refinement and Reduction of Animals in Research–NC3Rs, with due pre- and post-operative care with analgesic control measures and good aseptic practices for carrying out the procedures [70].

The healing process of critical defects in bone with mineral deficiency can be altered in order to stimulate heterotopic formation and stimulate the resorption of the edges of the defect [71]. In the radiological analysis, well-preserved contours of bone defects were observed, with radiopacity of the cortical margins of the tibia in all study groups, also ruling out bone rarefactions or infectious processes such as osteomyelitis. In the center of the bone defects, rounded areas with radiolucencies present and delimited by the cortical margins were observed. No secondary fractures, pseudarthroses, carcinogenic processes or any other morphological abnormalities were observed, as shown in image C of Figure 4.

### 2.3. Histomorphometric and Histomorphologic Analysis of Bone Defects

In recent years, the introduction of polymers into regenerative therapies has enabled significant advances in tissue engineering [46]. In addition to collagen, other natural polymers such as chitosan and fibroin and synthetic polymers such as polylactic acid (PLA) and polycaprolactone (PCL) have stood out for their biocompatibility and osteoconductivity [72]. However, each material has limitations that hinder clinical applicability. Chitosan and fibroin have poor osteoinductive and mechanical properties, causing instability in critical defects [73,74,75], while synthetic polymers have low biological activity and resistance [76,77]. Furthermore, a low absorption rate and hydrophobic characteristics make the adsorption of implants difficult, especially PLA, whose prolonged hydrolysis products create an acidic environment that induces local inflammation [76]. For this reason, these polymers have been combined with nHA to form scaffolds with better biological and osteoconductive potential and greater mechanical resistance [72].

As an alternative to the critical aspects identified with the application of some natural and synthetic polymers, this study tested the osteogenic and osteoconductive differentiation capacity of collagen matrices mineralized with nHA and added elastin, a protein that presents hydrophobicity, as well as synthetic polymers, which can confer greater resistance to the scaffold but without causing cytotoxic effects in vivo. The viability of the biomaterials was also evaluated in healthy rats and rats with gonadal deficiency caused by ovariectomy. Another differentiator of these scaffolds is the use of nanotechnology, with the aim of mimicking bone morphology. The stiffness of native bone is due to the deposition of nHA in gaps measuring 40nm formed by collagen fibers, and for this reason, nanostructured biomaterials have a clinical advantage [78]. Hydroxyapatite nanoparticles with a size of 1 to 100 nm present greater biological activity and enable the adhesion of mesenchymal stem cells [79], avoiding a drastic increase in the rigidity and resistance to degradation of the scaffolds [80]. This characteristic was interesting mainly for MSHC matrices, in which a greater hardness of the biomaterial was already expected due to the presence of elastin.

The characterization of the MSH and MSHC blends demonstrated that the alkaline hydrolysis and freeze-drying process generated average porosity of 28.8 and 26.1 µm, where the pores remained interconnected and nucleated by the nHA particles inside, preserving the three-dimensional architecture for the absorption of fluids and the possibility of stimulating angiogenesis. Busquets et al. (2016) describe that microporosity has the bonus of stimulating cell adhesion and the burden of compromising vascular permeability, hindering gas exchange and the removal of metabolic products. Contrasting this with the smaller diameter of the pores of the MSHC matrix used in this study, they did not harm the bone-defect repair process [81].

Regarding the nanotopography of the scaffolds used, calcination at a temperature of 550 °C was a positive strategy for obtaining hydroxyapatite nanoparticles. Normally, the method of obtaining and purifying synthetic hydroxyapatite requires calcination at high temperatures, which can cause the loss of carbonate, the formation of oxide and a change in the composition and morphology of calcium phosphate crystals [82]. Studies indicate that temperatures above 700 °C favor the appearance of β-tricalcium phosphate (β-TCP) and the change from nanocrystals to microcrystals—factors that can influence clinical results due to compromising the porosity of the scaffold [83,84].

**Figure 5:**
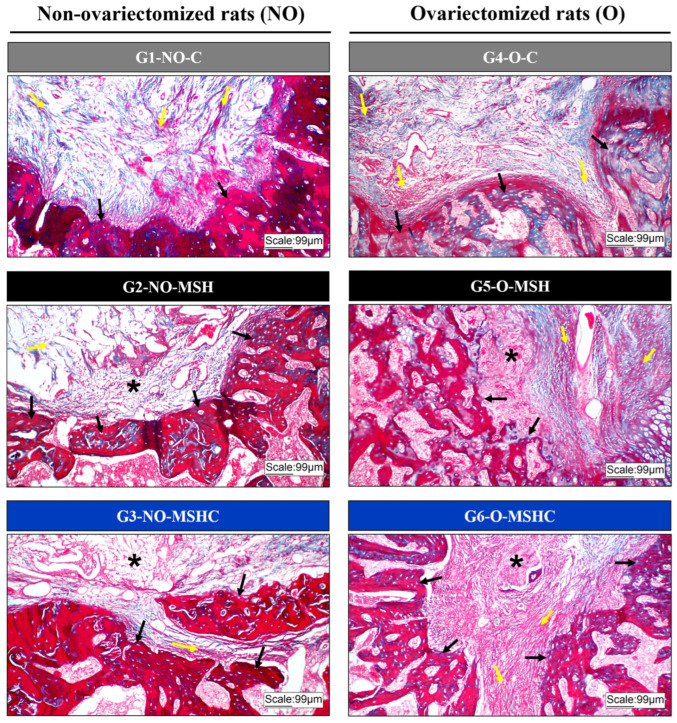
Histological image with Masson’s trichrome of the non-ovariectomized groups on the left (G1-NO-C, G2-NO-MSH and G3-NO-MSHC) and ovariectomized groups on the right (G4-O-C, G5-O-MSH and G6 -O-MSHC). Black arrows indicate foci of new bone formation; Yellow arrows indicate the presence of loose connective tissue; Asterisk (*) indicates the presence of scaffolds. 10× increase.

Despite the addition of elastin in MSHC, both scaffolds showed similar bone formation, both in healthy animals and in animals with bone-mineral deficiency. In contrast to expectations, the addition of a hydrophobic protein did not provide greater resistance to the matrices in in-situ absorption tests, which showed a higher degradation rate than MSH. The same result was confirmed in vivo by the presence of smaller fragments of the elastin-containing scaffolds in the bone defects. In contrast to the more accelerated biodegradation of the MSHC scaffold, intense osteogenic differentiation was also observed, especially in the healthy group (G3-NO-MSHC), in which it was possible to observe bone neoformation with a more compact morphology; the bone was rich in osteocytes and had maturity characteristics, confirmed by the predominance of the red color in slides stained with Masson’s trichrome. However, in the group with ovariectomy (G6-NO-MSHC), the new bone was more trabeculated, containing osteocytes and with hematopoietic tissue interposed between the trabeculae; it had characteristics of immaturity due to the bluish color found in the histological slides (Figure 5).

**Figure 6:**
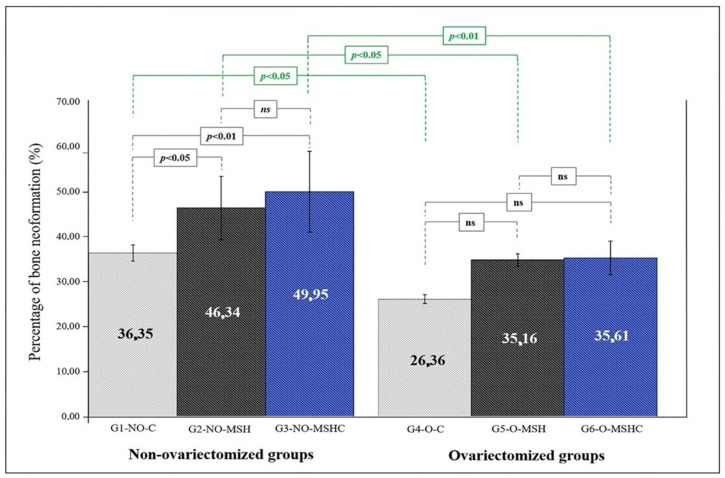
Analysis of the percentage of new bone formation in the healthy and ovariectomized groups. (ns) Not significant.

MSH and MSHC membranes offered support for osteoconduction in all experimental groups since in histology it is possible to observe that the neoformation started at the edges of the defect and migrated to the center of the bone lesion. The MSHC matrix, when implanted in defects of healthy bone (G3-NO-MSHC), offers superior and significant osteoconductive potential compared to the healthy control group (G1-NO-C) and the homologous group with estrogen deficiency (G6-O-MSHC). However, the biomaterial with elastin was not significantly better than MSH in terms of the rate of bone neoformation. This result was verified in the statistical comparison between the healthy groups and between the sick groups (Figure 6).

It is established in the literature that mineralized collagen offers advantages to the cellular chemotaxis process [85,86], in addition to providing greater longevity to the scaffolds [25]. The inclusion of proteins and growth factors also represents a promising alternative, but errors in dosimetry are associated with osteolysis, ectopic bone formation and carcinogenesis. These clinical problems have already been identified with the use of bone morphogenetic protein (BMP), parathyroid hormone (PTH) and platelet-derived growth factor (PDGF) [87]. The absence of toxicity and immunogenicity are also decisive factors for the success of regenerative therapies guided by biomaterials. In the case of the rejection of a foreign body, neutrophils are recruited and secrete protelytic and oxygen-reactive enzymes, releasing chemokines that attract immune cells such as monocytes, which later differentiate into macrophages. The latter release pro-inflammatory factors, attracting giant cells and causing chronic local inflammation [88,89].

None of the scaffolds applied in vivo in this study demonstrate signs of immunological rejection due to the absence of giant cells and the encapsulation of the scaffolds by fibrous tissue. Especially in those with bone-mineral deficiency (G5-O-MSH and G6-O-MSHC), the interposition of loose connective tissue was present between the remnants of the membranes and the newly formed bone lamellae; however, it did not present encapsulation characteristics. However, better osteogenic results were expected for the collagen matrix from porcine serosa that was mineralized with nHA and supplemented with elastin from bovine auricular cartilage.

One explanation for the similar results found between MSH and MSHC lies in the important capacity of collagen in the process of activating the enzymatic signaling pathways for extracellular regulated protein kinase (ERK), protein kinase (PKB) and phosphatidylinositol kinase (PI3K), which stimulate the osteogenic differentiation of mesenchymal cells [90]. It also has affinity for leukocyte-associated immunoglobulin-like receptor 1 (LAIR-1), which through its two immunoreceptor tyrosine-based activation motifs (ITAMs), causes the phosphorylation of the Syk, zap70 and PLC-γ domains, and thus, there is a barrier to the action of protein kinases and recruitment of protein tyrosine phosphatases I and II. In this way, osteoclastogenesis mediated by LAIR-1 is inhibited, preventing bone resorption [71,72,73,74,75,76,77,78,79,80,81,82,83,84,85,86,87,88,89,90,91].

The lower osteogenesis analyzed in all groups with gonadal deficiency may be related to changes in mineral metabolism, such as in the case of magnesium, which has important implications for the cell adhesion process; zinc, which acts as a stimulating agent for osteoblast differentiation; and potassium in the biochemical control and apatite deposition [19]. One study incorporated magnesium ions into an intrafibrillar collagen and HA scaffold, obtaining significant results for osteoinductivity [92,93].

The biomaterials analyzed in this study offer support for cell proliferation, even under unfavorable osteometabolic conditions caused by ovariectomy. This is an important finding and indicates the ECM’s ability to interact and mimic. Picrosirius-red histological staining allowed for the analysis of the formation, density and orientation of collagen fibers. In healthy groups, the collagen fibers of the newly formed bone tissue were marked predominantly in red, with dense clustering indicating the formation of compact bone. It was also possible to observe the collagen fibers of the connective tissue presenting the same color pattern, but they were more spread out and in parallel, migrating in multiple directions (loose and disorganized connective tissue).

**Figure 7:**
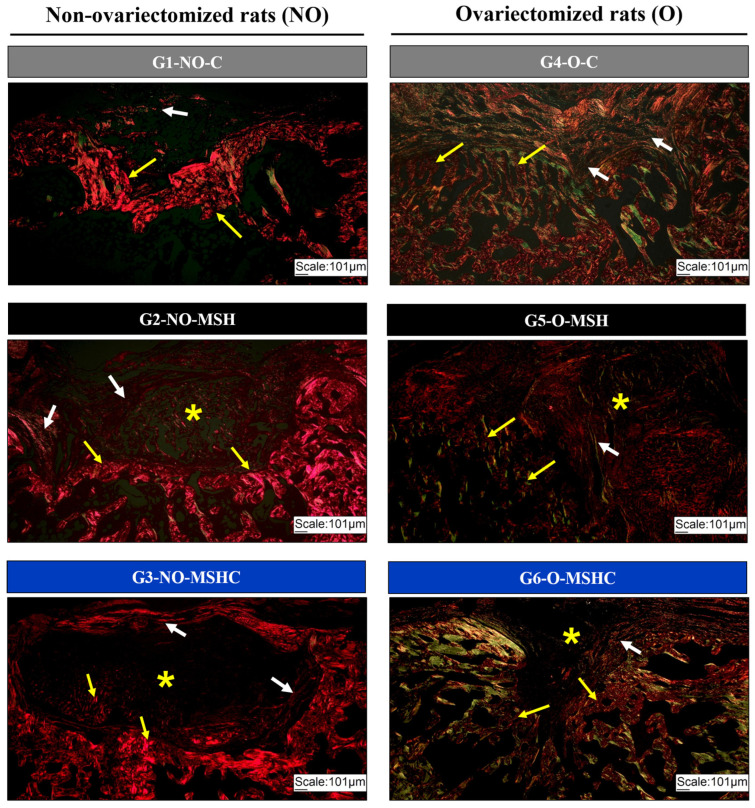
Histological image with Picrosirius-red of the non-ovariectomized groups on the left (G1-NO-C, G2-NO-MSH and G3-NO-MSHC) and ovariectomized groups on the right (G4-O-C, G5-O-MSH and G6- O-MSHC). White arrows indicate foci of new bone formation; Yellow arrows indicate the presence of loose connective tissue; Asterisk (*) indicates the presence of scaffolds. 10× increase.

In groups with gonadal deficiency, collagen fibers showed birefringence to polarized light, with a color pattern in orange and greenish tones, present in bone neoformations and in areas with interposed connective tissue. Sick animals showed a lower formation and density of collagen fibers (Figure 7).

Considering that natural polymers composed of proteins present potential characteristics for osteoregeneration, contributing to our results, Hejazi and collaborators analyzed the capacity for bone regeneration guided by scaffolds composed of elastin, polycaprolactone and nanohydroxyapatite that were seeded with mesenchymal stem cells and implanted in the femurs of healthy and ovariectomized rats. Five weeks post-surgery, the experimental groups showed a significant increase in stereological parameters, with a greater expression of BMP and VEGF [35]. Rodrigues et al. (2021) describe that the association of elastin with collagen enabled better cell adhesion [94]. 

This research represents the continuation of a line of research in bone tissue regenerative therapies supported by scaffolds derived from natural polymers. In a previously carried out study, collagen/chitosan/hydroxyapatite polymeric membranes were produced using two different methods for the deposition of calcium phosphate crystals (in-situ and ex-situ); they were implanted in experimentally caused bone defects in the tibia of healthy rats and in rats with hormonal deficiency induced by ovariectomy. Both scaffolds showed biocompatibility and osteoregenerative potential; however, the scaffolds produced with the ex-situ deposition of calcium phosphate ions allowed for better results in healthy animals [5]. Another study applied supports to the femur of rats that were made with auricular elastin, treated with different temperatures and times of alkaline treatment, and supplemented with hydroxyapatite and BMP. The most important finding was determining the elastin treatment time, which is crucial for maintaining ideal porosity after HA deposition. The scaffold processed for 96 h presented more favorable porosity for cell permeability compared to the scaffold processed for 24 h [95]. Recently, Pandini et al. (2022) separately determined the osteoconductive potential of three polymers (collagen, elastin and chitosan) in healthy rats and rats with metabolic deficiency caused by alcohol ingestion. The highest rates of bone neoformation occurred with chitosan scaffolds, followed by elastin and collagen, respectively. The same was observed in animals subjected to alcoholism [18].

Among the limitations of the present study are the restriction on the number of animals and the absence of other experimental groups, in which the effects of each composite could be tested separately and under different processing conditions. The importance of further investigations of natural components is highlighted for the development of more efficient scaffolds with controlled biodegradability, bioactivity and mechanical resistance, with the aim of providing support for tissue growth throughout the process of repairing serious bone defects.

## 3. Materials and Methods

### 3.1. Biomaterials

The scaffolds were prepared and characterized by master’s student Claudio Fernandes Garcia in the Biochemistry and Biomaterials Group of São Carlos Institute of Chemistry–USP. The materials used were collagen obtained from porcine serosa, bovine ear cartilage and synthesized nanohydroxyapatite.

### 3.2. Collagen Preparation

The anionic collagen was obtained from porcine serosa, purchased from a meat store. The serosa was cleaned with water, cut, and washed in 0.5% acetic acid and 0.5% sodium hydroxide solutions, to remove fat and blood. It subsequently underwent alkaline hydrolysis [55] in solution with hydroxides, chlorides and sulfates of K+, Ca_2_ + e Na+ for 120 h. This solution promotes the selective hydrolysis of carboxyamide groups of the collagen, increasing the negative charge of the biopolymer. Subsequently, all salts were removed by washing in 3% boric acid solution, deionized water and 0.3% EDTA and again with deionized water until pH 6.0. The material was lyophilized and solubilized in acetic acid, pH 3.5, to obtain a 4% collagen solution.

### 3.3. Nanohydroxyapatite (nHA) Synthesis

For the synthesis of nHA, 100 mL of 0.01 mol L^−1^ of cetrimonium bromide (CTBA) was slowly added to 0.6 mol L^−1^ of K_2_HPO_4_. The pH was adjusted to 12 with NaOH and the mixture was stirred for 2 h. After, a 1.0 mol L^−1^ CaCl_2_ solution was added, under constant stirring. The suspension formed was placed under reflux for 6 h and then ultrasonicated for 1 h and washed with deionized water and ethanol. Afterwards, the precipitate was placed at 40 °C for 12 h to evaporate the solvent and then calcined at 550 °C for 5 h. The characterization of hydroxyapatite is presented in Garcia et al. (2021) [96], with a crystalline grain size of 38.9 nm and a crystallinity percentage of 80.4%.

### 3.4. Bovine Ear Cartilage Treatment

Bovine ear cartilage was first cut and washed with 0.9% NaCl solution to remove blood and other contaminants. After that, it underwent an alcaline hydrolysis treatment [55] as described for the preparation of collagen but for a period of 15 h at 37 °C. The resulting material was suspended in acetic acid solution, pH 3.5, and lyophilized.

### 3.5. Scaffold Preparation

Two kinds of scaffolds were prepared: porcine serosa collagen with nanohydroxyapatite (MSH) and porcine serosa collagen with nanohydroxyapatite and bovine ear cartilage (MSHC). At first, the 4% collagen gel was diluted to 1.5% with a suspension of nanohydroxyapatite in HAc pH 3.5 using the ratio of 30 mg of nHA for every 10 g of 1.5% collagen gel; it was used to prepare MSH by stirring at 900 rpm. Scaffolds containing bovine auricular cartilage (MSHC) were made by adding 50 mg of cartilage for every 10 g of collagen/nHA gel. The scaffolds were obtained by freeze-drying 6g of each preparation in 11 × 1.5 cm Teflon^®^ molds. The obtained scaffolds were neutralized in ammonia vapor for a period of 2 h and then aerated under air flow for at least 72 h.

### 3.6. Scaffolds Characterization

#### 3.6.1. Scanning Electronic Microscopy (SEM)

Analysis was carried out with the scaffolds glued to stubs using conductive carbon tape, previously covered with a thin 6 nm thick layer of gold, in a metalizer Coating System BAL-TEC MED 020 (BAL-TEC^®^, Liechtenstein) with a pressure in the chamber of 2.00 × 10^−2^ mbar, a current of 60 mA and a deposition rate of 0.60 nm/s, enabling the analysis of their structures. ZEISS^®^ LEO 440 (Cambridge, UK) equipment was used with an OXFORD^®^ (model 7060) detector, operating with a 20 kV electron beam. The pore size was obtained using Image J software using 30 determinations at 500× magnification. An approximation of the Martin diameter was used [97].

#### 3.6.2. Differential Scanning Calorimetry (DSC)

The thermal stability of scaffolds was determined by differential scanning calorimetry (DSC-2010^®^, TA Instruments) from 5 to 120 °C. Heating was performed in hermetic aluminum pans in a nitrogen atmosphere (80 mL min^−1^) at a rate of 10 °C min^−1^ using 20 mg of sample. The temperature of denaturation was given from the inflection point of DSC curves.

#### 3.6.3. Phosphate-Buffered Saline (PBS) Absorption

Scaffolds were placed in a chamber containing NaOH for 24 h and then weighed. After that, the scaffolds were placed in phosphate-buffered saline (PBS). At specific time intervals, they were removed, and the excess of solution was removed using a 2 cm × 2 cm filter paper. The scaffolds were weighed and returned to PBS for the further time taken. The percentage of PBS absorbed was calculated by averaging the results found using Eq. (1), where whumid is the mass of the swollen scaffold and wdry is the mass of the scaffold before swelling. The process was carried out in quadruplicate.

(1) $$\mathrm{absorption}\;\left(\%\right)=\frac{\mathrm{whumid}-\mathrm{wdry}}{\mathrm{wdry}}\;\cdot \;100$$

### 3.7. Experimental Design

This study was approved by the Animal Experimentation Ethics Committee of the Faculty of Medicine of Jundiaí (CEUA/FMJ–Brazil), protocol 246/2019, and included sixty female Wistar rats (Rattus norvegicus), 20 weeks old and with an average weight of 350 g. This work followed the standards of the National Council for the Control of Animal Experimentation (CONCEA) and kept the animals in a vivarium with a controlled temperature (23 ± 1 °C) and a 12/12-h light/dark cycle, receiving balanced food (Purina^®^) and water ad libitum.

**Figure 8:**
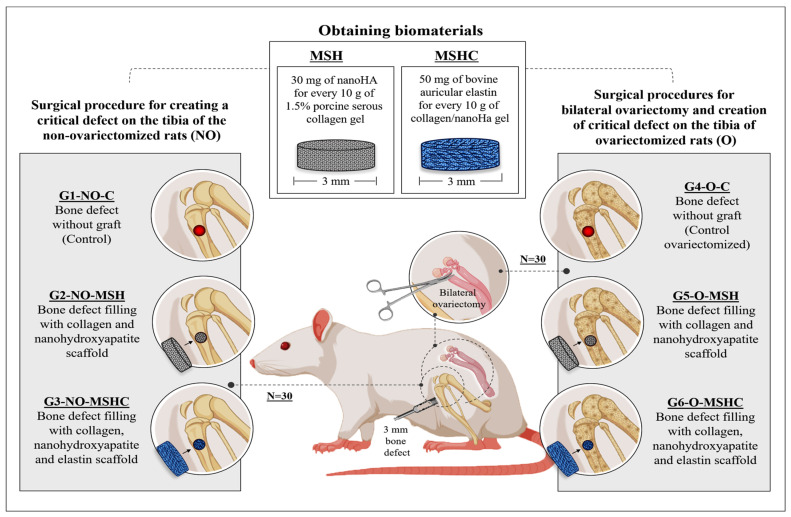
Experimental design after obtaining the biomaterials with the distribution of study groups and respective surgical procedures and scaffolds.

The sample was divided into three non-ovariectomized healthy groups (G1-NO-C; G2-NO-MSH; G3-NO-MSHC) and another three bilaterally ovariectomized groups to stimulate estrogen deficiency, and consequently, a reduction in bone-mineral density (G4-O-C; G5-O-MSH; G6-O-MSHC), according to the distribution described in Table 3 and Figure 8.

### 3.8. Surgical Procedures

**Figure 9:**
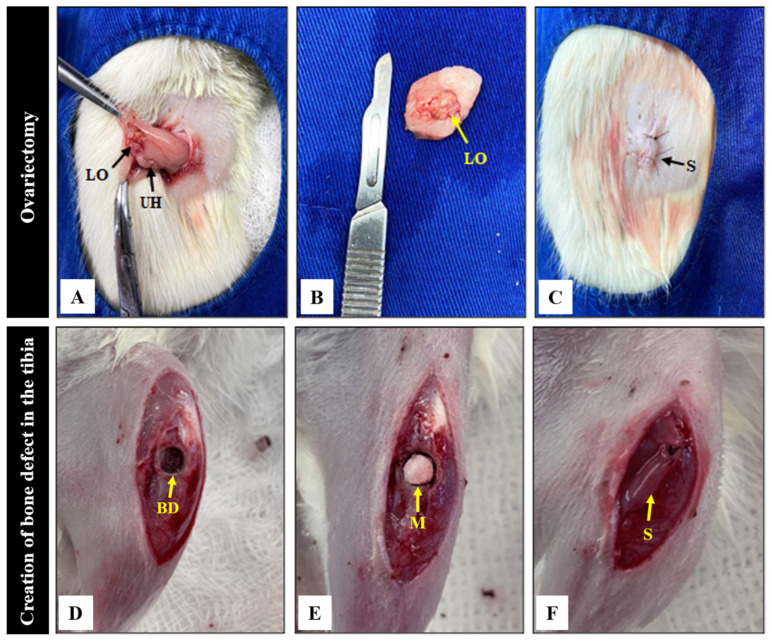
Bilateral ovariectomy of rats in groups 4, 5 and 6 and procedure to create the bone defect in the tibia of groups 1 to 6. (**A**) Location of the uterine tube (UH) and left ovary (LO); (**B**) Surgical resection of the left ovary with portion of the uterine tube; (**C**) Repositioning of the uterus, suture performed in planes on the left side (S); (**D**) Surgical approach to the left tibia and creation of the bone defect (BD); (**E**) Grafting of groups 2, 3, 5 and 6 with polymeric membrane (M); (**F**) Suture of the periosteum and leg muscles for subsequent skin closure. Note: Ovariectomy was performed bilaterally.

At 12 weeks of age, the animals in the ovariectomized groups (G4-O-C; G5-O-MSH; G6-O-MSHC) were weighed and anesthetized with a xylazine solution at 1 mg/kg (Vetaset^®^) (a sedative, analgesic and muscle relaxant) and ketamine (Dopalen^®^) at a dose of 70 to 80 mg/kg (an anesthetic agent with sedative effect), and both were applied intramuscularly in a 1:1 ratio [98]. Tramadol was also administered subcutaneously at 10 mg/kg (2% Cronidor^®^, Brazil) as a post-operative pain control measure [99]. After the trichotomy and asepsis of the dorsal region with 2% chlorhexidine digluconate, a 2 cm skin incision was made on both sides of the spine. The subcutaneous tissue was detached to visualize the local muscles. It was sectioned in the longitudinal direction, giving access to the retroperitoneum. In this way, the ovaries were identified and removed through the section and surgical removal of the uterine lobes and fallopian tubes [100,101] (Figure 9).

After surgery, the tissues were sutured with nylon thread (Ethicon^®^, Johnson and Johnson, São José dos Campos, Brazil) and each animal received an intramuscular dose (0.1 mg/100 g) of antibiotic (Pentabiotic Veterinário Pequeno (Fort Dodge^®^, Campinas, Brazil). At the site of the skin wound in the surgical region, antibiotic rifamycin spray (Rifotrat^®^, Brazil) was applied as a second measure to control post-operative pain. The surgical approach was adopted with the dilution of paracetamol (15 drops) in water pipettes [60].

At 20 weeks of age, all animals in the healthy non-ovariectomized groups (G1-NO-C; G2-NO-MSH; G3-NO-MSHC) and in the ovariectomized groups (G4-O-C; G5-O-MSH; G6- O-MSHC) underwent a surgical procedure to create a bone defect in the left tibia. 

The animals were positioned in the supine position for the shaving and asepsis of the left hind paw. A longitudinal skin incision was made to expose and separate the proximal muscles of the left leg. Once the proximal metaphysis of the left tibia was exposed, the periosteum was detached and a bone defect of 2.5 mm in diameter was created with a trephine drill coupled to the pen of a minimotor (Beltec LB-100^®^, Brazil), with continuous irrigation until reaching the spinal canal, of which the hematopoietic content was aspirated and washed with 0.9% saline. The bone defects in groups 2, 3, 5 and 6 were grafted with polymeric matrices, while control groups 1 and 4 remained empty with the defect. Then, the soft tissues, including the periosteum, muscles, fascia and skin, were sutured. The tibia procedure followed the same operative care protocol, including anesthesia, antisepsis, antibiotic therapy and analgesia.

### 3.9. Macroscopic, Radiological and Histomorphometric Analysis In Vivo

Six weeks after the procedure on the tibia, the animals were sacrificed by an overdose of intramuscular anesthetic, followed by pneumothorax. The left tibia was carefully dissected and removed, preserving the soft tissue over the bone defect area. After collecting photodocumentation to analyze clinical conditions, the tibias were X-rayed to evaluate the integrity and radiodensity of the experimental area. They were then kept in a formaldehyde solution for 15 days and were subsequently decalcified in ethylenediaminetetraacetic acid (EDTA) and reduced transversely, preserving the defect area.

The samples were processed by histology with semi-serial sections of 5 µm, stained with Masson’s trichrome to characterize new bone formation, and stained with Picrosirius-red (saturated aqueous solution of picric acid plus 0.1 g of Sirius red F3B, Bayer^®^, Germany) for the identification of the components of the fibrillar extracellular matrix by polarized light microscopy. For the qualitative histological analysis, several aspects of the local microenvironment were observed, such as cell types, presence of granulation tissue, presence and quality of immature or mature/lamellar bone, inflammatory infiltrate and the degree of filling of the newly formed tissue.

For morphometry, three semi-serial sections were analyzed and stained with Masson’s trichrome, obtained from the proximal, median and distal region of the bone defect area of each sample, obtaining three data points on the volume of newly formed bone per animal for the subsequent calculation of new training averages per study group. The quantification of new formation was carried out using the Motic Images Plus 2.0 ML software to delimit the total area of the bone defect and newly formed bone. The quantified volumes for the samples from each group were transcribed into the BioEstat 5.3 software, and statistical analysis was conducted using the ANOVA test followed by the Tukey test, with a confidence interval of 95% and a significance level of *p* < 0. 05, to determine the differences between the groups.

## 4. Conclusions

This study aimed to evaluate the osteoregenerative capacity of porcine serous collagen scaffolds mineralized with nanohydroxyapatite, supplemented or not with elastin derived from bovine auricular cartilage, used as a therapeutic resource for guided regeneration in size defects created in the tibia of healthy rats and rats with bone-mineral deficiency induced by ovariectomy. Macroscopic, radiological and histomorphometric analyses revealed the formation of new organized and mature bone tissue, the absence of intense inflammatory reaction or foreign body reaction, and osteoconductivity properties of both matrices (MSH and MSHC), in animals from groups with normal conditions of osteometabolism and groups with gonadal deficiency. The scaffolds demonstrated a higher percentage of bone formation when compared to the control groups, and there was no significant difference in the rate of new bone formation between the scaffolds. Ovariectomy did not exacerbate the immune response but negatively influenced the bone-defect repair process.

## Figures and Tables

**Table 1:** Scaffolds pore sizes.

Scaffold	Pore Size (μm)		
	Smaller	Bigger	Mean ± SD
MSH	14.3	48.7	28.8 ± 7.8 ^a^
MSHC	13.9	39.7	26.1 ± 5.2 ^a^

In the same column, equal letters mean statistically equal values (*p* < 0.05). SD: standard deviation.

**Table 2:** PBS absorption (%).

Scaffolds	Absorption (%)		
	30 min	60 min	1440 min
MSH	745 ± 158 ^a^	886 ± 276 ^a^	2291 ± 105 ^a^
MSHC	2566 ± 136 ^b^	2574 ± 187 ^b^	2837 ± 158 ^b^

In the same column, equal letters mean statistically equal numbers (*p* < 0.05).

**Table 3:** Experimental groups, with their respective fillings in the surgically created bone defect.

	Non-Ovariectomized Rats (NO)	Ovariectomized Rats (O)	Without Graft (only Clot)	Collagen with nHA Scaffold	Collagen with nHA and Elastin Scaffold
G1-NO-C	X		X		
G2-NO-MSH	X			X	
G3-NO-MSHC	X				X
G4-O-C		X	X		
G5-O-MSH		X		X	
G6-O-MSHC		X			X

## Data Availability

The data presented in this study are available on request from the corresponding author.
